# Neuropsychological Test Norms for the Assessment of HIV-Associated Neurocognitive Impairment Among South African Adults

**DOI:** 10.1007/s10461-023-04029-9

**Published:** 2023-03-14

**Authors:** Melanie Deist, Sharain Suliman, Martin Kidd, Donald Franklin, Mariana Cherner, Robert K. Heaton, Georgina Spies, Soraya Seedat

**Affiliations:** 1 South African PTSD Research Programme of Excellence, Department of Psychiatry, Stellenbosch University, Cape Town, South Africa; 2 South African Medical Research Council/Stellenbosch University Genomics of Brain Disorders Research Unit, Department of Psychiatry, Stellenbosch University, Cape Town, South Africa; 3 Centre for Statistical Consultation, Stellenbosch University, Stellenbosch, South Africa; 4 The HIV Neurobehavioral Research Center (HNRC), San Diego, USA

**Keywords:** Neurocognitive testing, Norms, South Africa, HIV, HAND

## Abstract

Reliable and valid neurocognitive (NC) test batteries that assess multiple domains of cognitive functioning are vital tools in the early detection of HIV-associated NC impairment. The HIV Neurobehavioral Research Center’s International Neurobehavioral Battery (HNRC Battery) is one such diagnostic tool and has shown cultural validity in several international neuroHIV studies. However, no published norms are currently available for the full HNRC Battery in South Africa. To accurately interpret NC test results, appropriate reference norms are required. In light of this challenge, data were collected from 500 healthy, HIV-uninfected participants to develop demographically corrected South African norms. When demographically corrected United States of America (U.S.) norms were applied to the performance scores of our neurologically intact, HIV-negative sample, an impairment rate of 62.2% was observed compared to a 15.0% impairment rate when the newly generated South African norms were applied. These results reiterate the findings of other low- and middle-income countries, highlighting the need for localized, country-specific norms when interpreting NC performance.

## Introduction

The human immunodeficiency virus (HIV) can induce neurocognitive (NC) impairment [1–4]. The umbrella term for the spectrum of NC disorders that present in people with HIV (PWH) is HIV-associated neurocognitive disorders (HAND) [5–7]. Even though advances in antiretroviral treatment have dramatically decreased the incidence of more severe forms of HAND, the incidence of mild NC impairment persist among PWH [6–10].

Milder forms of HAND have been associated with impaired instrumental activities of daily living, employment difficulties, and a worse overall quality of life. Escalating degrees of NC impairment are also associated with higher mortality rates, lower adherence to complicated treatment regimens, and poorer health-related decision-making [11–18]. Given the functional consequences of HAND, it is vital to identify early signs of NC impairment as soon as possible. Identifying early signs of NC impairment aids in the long-term clinical management of HAND, the initiation and adjustment of treatment regimes, and the monitoring of disease progression and treatment effects [8, 19, 20]. Moreover, diagnosing HAND early provides the opportunity for additional neuroprotective and psychosocial therapies that minimise NC decline, improve cognitive reserve, and ultimately improve the quality of life [5, 8, 21].

South Africa is at the epicentre of the global HIV epidemic [22]. Therefore, it is imperative that culturally sensitive tools are identified to facilitate early detection of HAND in this vulnerable population. The HIV Neurobehavioral Research Center’s International Neurobehavioral Battery (HNRC Battery) is a comprehensive assessment tool sensitive to the NC effects of HIV [23]. It was initially developed for use in research settings and has been used successfully in South African HIV studies [24–26]. The HNRC Battery conforms to the Frascati recommendations published by Antinori et al. [5] and is sensitive to both cortical and subcortical patterns of NC impairment [27]. The extensive battery could be a viable option to aid in the early detection of HAND in the South African context [23]. However, like other extensive NC tests, the HNRC Battery present various challenges in resource-limited settings. First, the administration of the HNRC Battery is time consuming, taking an average of two hours to complete. This is problematic for resource-limited settings, like South Africa, that are often faced with time constraints and a lack of local expertise. Second, to our knowledge, no South African norms are currently available for this battery [25]. If South African norms are made available, an abbreviated version of the HNRC Battery could have utility in both research settings (i.e., for studies on HAND in SA) and clinical settings (e.g., regional, and tertiary hospitals where referral to a neuropsychologist for further evaluation, when indicated, is possible).

Norms can be defined as the performance of a well-defined population that provide an empirical frame of reference for determining which test scores are “normal” or “typical” at a specific time point [28]. Different cultural environments emphasise or de-emphasise differing abilities based on ecological demands and situational relevance, which may impact on performance, administration, and interpretation of NC measures [19, 29–33]. Therefore, cultural aspects are important considerations when determining what constitutes a “normal” or “typical” NC test performance [5, 19, 33–38]. Without culturally appropriate norms, test scores derived from the HNRC Battery may result in significant errors in diagnosis (false positives and negatives) [19, 29, 33, 38, 39]. The present study sought to address this limitation by developing demographically corrected neuropsychological norms for the HNRC Battery in the South African context.

## Methods

### Study Design and Setting

This study was nested within a larger study that sought to enhance the practicality of the HNRC Battery for use in South African clinics. The study consisted of three distinct phases. The first phase entailed the development of demographically corrected South African norms for the HNRC Battery, which is described in this paper. These norms were then used in the second and third phases, which involved the development and validation of an abbreviated version of the HNRC Battery (see Spies et al. [40]). The study was cross-sectional in design and data collection ran from May 2016 to June 2019.

### Recruitment

Using convenience sampling, HIV-negative South African adults were recruited from the Cape Metropolitan and Winelands areas in the Western Cape of South Africa. In addition, the study made use of secondary data collected from an ongoing longitudinal, prospective study (Ethics reference number: N07/07/153) [26].

NC performance can be influenced by several confounding factors, which hinder the validity of research focused on the effects of HIV on NC test performance [3, 19]. To ensure that the norms developed were not influenced by confounding variables, the present study used exclusion criteria consistent with prior NC norming studies in low- and middle-income countries (LMICs) (e.g., [41–47]). Specifically, participants were excluded if they met the following criteria: (a) a history of neurological disease (e. g. dementia, seizure disorders); (b) severe head injury resulting in loss of consciousness for more than 30 min; (c) prior neurosurgery; (d) a history of psychotic disorders; (e) current anxiety and mood disorders or high suicidality (as measured by the Mini International Neuropsychiatric Interview 7.0 [MINI 7.0] [48, 49]; (f) post-traumatic stress disorder (PTSD); (g) a history of learning disorders (e.g., dyslexia) or Attention Deficit/Hyperactivity Disorder (ADHD); (h) past or current chronic use of psychotropic medication; (i) current severe alcohol use disorder; (j) regular cannabis use in the last six months; and (k) drug abuse in the last 2 years, excluding cannabis. To ensure that participants were able to read and understand the informed consent documents and complete the neurocognitive assessment, the following exclusion criteria applied: (l) an inability to read or write in either English, Afrikaans, or isiXhosa; and (m) formal education of fewer than 7 years.

The final sample included 500 volunteers who tested negative for HIV infection using a Rapid HIV-1 blood Test. Participants completed the HNRC Battery in the three official provincial languages of the Western Cape [50]: English (*n* = 200), isiXhosa (*n* = 150), or Afrikaans (*n* = 150). Each participant received a shopping voucher to the value of ZAR100 (about 7$USD at the time of the study) as a token of gratitude. Travel costs to the university were also reimbursed.

### Procedure

Ethical clearance was obtained from the Health Research Ethics Committee of the Faculty of Medicine and Health Sciences of Stellenbosch University (reference number: S15/05/124).

**Fig. 1:**
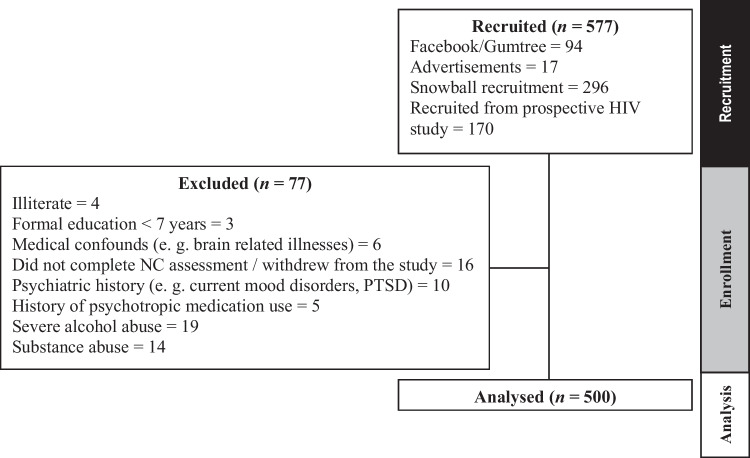
Recruitment process flowchart. *NC* neurocognitive; *PTSD* posttraumatic stress disorder

Participants were recruited using four approaches: (1) advertisement on social media platforms (i.e., www.gumtree.co.za and www.facebook.com); (2) flyers posted on notice boards in shops, churches, and clinics; (3) an advertisement in a local community newspaper; and (4) snowball recruitment. The recruitment process is outlined in Fig. 1.

Potential participants were initially screened for eligibility. Eligible participants were invited for assessment. Data were collected face-to-face in a once-off session in a private office on campus. Participants were fully briefed on the study details and provided written informed consent. Demographic characteristics (e.g., age, sex, race, and years of education) were captured using a self-report questionnaire. Participants were screened for current and lifetime psychiatric disorders using the M.I.N.I. 7.0 [48, 49]. The HIV status of each participant was confirmed via a Rapid HIV-1 blood Test. Confirmation of HIV status, coupled with pre- and post-test counselling, was either conducted at a government clinic specialising in family planning or by qualified study staff on-site.

NC test administration was conducted by researchers (a doctoral student and a professional research nurse), who received standardised training in the administration and scoring of the HNRC Battery. Training was conducted at Stellenbosch University in face-to-face meetings and included several rounds of supervised “mock testing” and interrater reliability sessions. To ensure consistency across assessments, the battery administrators had to follow a structured instruction manual verbatim during each assessment. Test administrators were regularly monitored during the study. Training was provided by a research psychologist, who was previously trained and certified in the administration and scoring of the HNRC Battery at the HNRC, University of Californian, San Diego.

### Neurocognitive Measures

The HNRC Battery typically takes 2–2.5 h to complete and is available in English, Afrikaans, and isiXhosa. Instructions for the NC battery were translated to Afrikaans and isiXhosa using standard techniques of forward and back translation. The battery consists of 17 individual test measures that evaluate seven cognitive domains known to be susceptible to the effects of HIV, i.e., learning, delayed recall, processing speed, attention/working memory, executive function, verbal fluency, and motor ability [3, 4, 7].

#### Learning and Delayed Recall

Immediate recall, learning rate, and delayed recall were measured using the Brief Visuospatial Memory Test-Revised (BVMT-R) [51] and the Hopkins Verbal Learning Test-Revised (HVLT-R) [52].

We used a modified version of the HVLT-R, adapting some of the semantic categories included in the original test to be culturally appropriate to the South African context. Precious stones such as “emerald”, “sapphire”, and “opal” are less known in South Africa. Therefore, the precious stones category was replaced with vegetables (bean, lettuce, corn, and potato). The words were also translated into Afrikaans and isiXhosa [26, 30].

The BVMT-R has demonstrated good interrater reliability, with reliability coefficients of 0.97 for the three learning trials, 0.98 for total recall, and 0.97 for delayed recall [51]. Test–retest reliability coefficients ranged from 0.60 to 0.84 for trial one to three, respectively [51]. The BVMT-R also has established construct validity [51]. The HVLT-R has acceptable reliability, with test–retest coefficients of 0.74 for total recall and 0.66 for delayed recall [52]. Interform reliability were also established, showing equivalent performance between different forms in both learning and delayed recall [52]. Furthermore, the HVLT-R demonstrated acceptable discriminant validity [52].

#### Processing Speed

Information processing speed was measured using two sub-tests of the Wechsler Adult Intelligence Scale-III (WAIS-III): the Digit Symbol and Symbol Search tests [53], and the Trail Making Test A [54].

The WAIS-III has established good test–retest reliability coefficients (0.88 to 0.94) and face, content, criterion-related, and convergence validity [53]. The Trail Making Test A have shown test–retest reliability coefficients ranging from low (0.46) to high (0.94) [54].

#### Executive Function

The Colour Trails Test 1 and Test 2 [55], the Stroop Colour and Word Test [56], the computer version of the Wisconsin Card Sorting Test (WCST) [57], and the computerised version of the Halstead Category Test [58, 59] all measured executive function and abstraction [55–57].

Colour Trails Test 1 and Test 2 have demonstrated test–retest coefficients of 0.64 and 0.78, respectively. Content and convergent validity have also been established [55]. The Stroop test has established good test–retest reliability, with the three sub-tests obtaining reliability coefficients of 0.86, 0.82, and 0.73, respectively [56]. Test–retest reliability coefficients of the WCST ranged from 0.37 to 0.72 [57]. The Halstead Category Test has demonstrated high internal consistency (0.95) and a test–retest reliability ranging from 0.60 to 0.90 [57]. There is no difference in how the standard and computer versions of the Category Test is administered or how it is recorded. The instructions given to the examinee are identical, as are the experiences of the examinees [60]. Further, there seems to be no statistically significant differences between the standard and computer versions when measuring total error scores, sub-test error scores, or Neuropsychological Deficit Scale scores [61].

#### Attention/Working Memory

The Wechsler Memory Scale-III (WMS-III) Spatial Span sub-test [58] and the Paced Auditory Serial Addition Task (PASAT): 50-item Short Form [62] were used to measure attention, concentration, and working memory [58, 62].

The WMS-III Spatial Span sub-test has demonstrated adequate internal consistency, generalisability coefficients, and test–retest coefficients ranging from 0.70 to 0.79 [63]. The PASAT has demonstrated very good test–retest reliability (0.73 to 0.96) and high internal consistency (0.90) [62].

#### Verbal Fluency

The Controlled Oral Word Association Test (COWAT)—FAS, the Category Fluency Test—Animals, and the Action/Verb Fluency Test are language tests included in the HNRC Battery to measure different types of verbal fluency [57]. The Afrikaans and isiXhosa versions of the COWAT were adapted. Specifically, the letters “F”, “A”, and “S” were replaced with “I”, “B”, and “S” in the isiXhosa version and “L”, “B”, and “S” in the Afrikaans version. In the isiXhosa translation, the selection of replacement letters was based on rank ordering the frequency of words in both an English and an isiXhosa dictionary. The isiXhosa words with a similar rank order to that of the English words beginning with the letters “F”, “A”, and “S” were selected. The same approach was used for the Afrikaans version of the verbal fluency tests [26, 30]. The COWAT (FAS) has demonstrated high internal consistency (0.83) and test–retest reliability coefficients (0.74) [57].

#### Motor Ability

The Grooved Pegboard Test [64] evaluates fine motor coordination and fine motor speed for both dominant and non-dominant hands [64]. The Grooved Pegboard Test has demonstrated test–retest reliability coefficients ranging from 0.67 to 0.86 [64].

### Statistical Analysis

Data analysis was done using Statistica version 12 [65] and R software [66], in partnership with the HNRC and a statistician from the Centre for Statistical Consultation at Stellenbosch University.

First, a regression analysis was performed to identify demographic characteristics significantly affecting the raw NC scores for each test.

Next, prediction equations were generated using the “mfp” R package [67], and the “test2norm” R package [68]. In the first step of this normative procedure, the raw scores for each NC test were converted into normally distributed scaled scores (*M* = 10; *SD* = 3). A multiple fractional polynomial (MFP) method [69] was then used to generate predicted test scores for each participant. The demographic variables that accounted for significant variance in raw test scores (i.e., age, education, gender, race, and NC test language) were entered into this MFP model to control for the variance in NC performance accounted for by these characteristics. Next, residual scores were calculated by subtracting the predicted scaled scores of each participant from their respective scaled scores. The residual scores were then converted to demographically corrected T-scores (*M* = 50; *SD* = 10) [69].

Finally, the impairment rates based on norms developed in the United States of America (U.S.) and the impairment rates based on the newly developed South African norms were compared. African-American norms were used for the self-described Coloured and Black South African samples and Caucasian norms were used for the self-described White South African sample [70–73]. To estimate the severity of “impairment”, demographically corrected T-scores (as determined by the U.S. norms and newly developed South African norms, respectively) were converted to deficit scores. In an imaging study that compared the accuracy of NC impairment classification methods in an HIV sample (i.e., Global Deficit Score [GDS], Frascati, and Meyer methods), the GDS criteria successfully detected brain abnormalities in an HIV-infected sample, supporting the continued use of this method in determining HIV-associated brain abnormalities [74]. GDS scores were converted as follows: 0 (T-score ≥ 40) = normal cognition; 1 (T-score 35–39) = mild NC impairment; 2 (T-score 30–34) = mild-to-moderate NC impairment; 3 (T-score 25–29) = moderate NC impairment; 4 (T-score 20–24) = moderate-to-severe NC impairment; and 5 (T score < 20) = severe NC impairment. The GDS was determined by averaging the deficit scores across all tests. Global NC impairment was assigned to participants with a GDS ≥ 0.50. The U.S. GDS and South African GDS were compared by calculating the proportion of neurologically intact, HIV-negative individuals defined as “impaired” by each set of normative equations.

## Results

### Demographic Characteristics

A total of 500 HIV-negative participants (age range = 18 to 63 years, mean age = 31.2, *SD* = 10.9 years) were evaluated. Most participants were women (*n* = 398; 79.6%) and self-identified as Black (*n* = 301; 60.2%). Most participants (*n* = 285; 57.0%) self-reported not completing secondary school education (grade 12) (*M* = 11.0, *SD* = 1.9), were unemployed (n = 373; 74.6%), and lived on an annual household income of below R20 000 (*n* = 329; 65.8%). isiXhosa was the home language spoken by most participants (*n* = 285, 57.0%).

The HNRC Battery was administered in English (*n* = 200), isiXhosa (*n* = 150), and Afrikaans (*n* = 150). Participants could complete the test battery in their preferred language. Most participants who completed the English battery, did not list English as their native language (*n* = 158; 79.0%), but chose to take the battery in English—the primary language of learning and teaching (LoLT) in many South African schools [75, 76]. However, all participants who completed the battery in isiXhosa and Afrikaans, listed isiXhosa and Afrikaans as their native languages, respectively.

A convenience sampling method was used throughout the study and language groups were not matched. The demographic differences between the three language sub-groups were compared to identify significant differences between the groups. Linear variables were compared using analysis of variance (ANOVA) and significant differences in age (*F*(2, 497) = 18.68,* p* < 0.001) and education (*F*(2, 497) = 11.321,* p* < 0.001) were observed between language groups. Categorical variables were compared using Chi-Square tests of association. Significant differences between groups were found on gender (*X*^2^(2, 500) = 21.86,* p* < 0.001), home language (*X*^2^(2, 500) = 418.23,* p* < 0.001), race (*X*^2^(2, 500) = 458.06,* p* < 0.001), marital status (*X*^2^(2, 500) = 38.80,* p* < 0.001), household income (*X*^2^(2, 500) = 72.64,* p* < 0.001), employment (*X*^2^(2, 500) = 6.54,* p* = 0.038), and handedness (*X*^2^(2, 500) = 13.47,* p* = 0.001).

Table 1 presents the demographic characteristics of the sample by language.

**Table 1:** Demographic characteristics of the sample based on language of administration of the HNRC battery

Demographic variable	English (*n* = 200)			isiXhosa (*n* = 150)			Afrikaans (*n* = 150)			Total (*n* = 500)		
	#	%	*M (SD)*	#	%	*M* *(SD)*	#	%	*M* *(SD)*	#	%	*M*
Gender												
Male	58	29.0		14	9.3		30	20.0		102	20.4	
Female	142	71.0		136	90.7		120	80.0		398	79.6	
Age (years)												
Younger than 35	154	77.0		106	70.7		75	50.0		335	67.0	
35 and older	46	23.0		44	29.3		75	50.0		165	33.0	
Mean (Standard deviation)			28.7 (10.2)			30.4 (9.0)			35.5 (12.1)			31.2 (10.9)
Education												
Did not finish Gr.12	98	49.0		95	63.3		92	61.3		285	57.0	
Finished Gr.12	82	41.0		47	31.3		36	24.0		165	33.0	
Finished tertiary education	20	10.0		8	5.3		22	14.7		50	10.0	
Mean (Standard deviation)			11.5 (1.6)			10.9 (1.5)			10.6 (2.4)			11.0 (1.9)
Home language												
isiXhosa	135	67.5		150	100.0		0	.0		285	57.0	
English	42	21.0		0	.0		0	.0		42	8.4	
Afrikaans	11	5.5		0	.0		150	100.0		161	32.2	
Other	12	6.0		0	.0		0	.0		12	2.4	
Race												
Black	151	75.5		150	100.0		0	.0		301	60.2	
Coloured	29	14.5		0	.0		121	80.7		150	30.0	
White	20	10.0		0	.0		29	19.3		49	9.8	
Marital status												
Single	150	75.0		99	66.0		73	48.7		322	64.4	
Married/living with a partner	38	19.0		48	32.0		60	40.0		146	29.2	
Separated/divorced/widowed	12	6.0		3	2.0		17	11.3		32	6.4	
Household income												
Less than ZAR20 000	116	58.0		134	89.3		79	52.7		329	65.8	
ZAR20 000–ZAR100 000	44	22.0		14	9.3		46	30.7		104	20.8	
More than ZAR100 000	40	20.0		2	1.3		25	16.7		67	13.4	
Employment												
Yes	52	26.0		28	18.7		47	31.3		127	25.4	
No	148	74.0		122	81.3		103	68.7		373	74.6	
Handedness												
Right	183	91.5		148	98.7		147	98.0		478	95.6	
Left	17	8.5		2	1.3		3	2.0		22	4.4	

The 2017 General Household Survey reported that 12.1% of South Africans spoke Afrikaans in their own household; 76.3% of South Africans who self-identified as Coloured and 57.9% of South Africans who self-identified as White spoke Afrikaans at home. Less than 1% of South Africans who self-identified as Black African spoke Afrikaans in their households. IsiXhosa was spoken in 15.6% of South African households. IsiXhosa was mostly spoken in households with members that self-identified as Black African; less than 1% of South Africans who self-identified as Coloured or White spoke isiXhosa at home [87].

### Demographic Influences on Raw Scores

Age, education, gender, race, and testing language accounted for significant variances in raw test scores. The percentage of variance in raw test scores uniquely accounted for by each of these demographic variables are presented in Table 2.

**Table 2:** Percentage of Variance in Raw Test-Scores adjusted for Demographic Variables

Neurocognitive test	Age F-value (% variance)	Education F-value (% variance)	Gender F-value (% variance)	Ethnicity F-value (% variance)	Language F-value (% variance)
BVMT-R (Total)	***79.02 (11.75)	***38.46 (5.72)	1.97 (0.29)	***8.15 (2.42)	0.51 (0.15)
BVMT-R (Recall)	***59.05 (9.23)	***25.77 (4.03)	1.13 (0.18)	**5.28 (1.65)	0.78 (0.24)
HVLT-R (Total)	*6.29 (0.91)	***31.32 (4.52)	*6.45 (0.93)	***8.81 (2.54)	1.49 (0.43)
HVLT-R (Recall)	***11.0 (1.83)	***26.43 (4.39)	*6.26 (1.04)	*3.88 (1.29)	0.64 (0.21)
WAIS-III Digit Symbol	***101.83 (12.01)	***124.84 (14.72)	***14.24 (1.68)	***27.42 (6.47)	*4.22 (1.00)
WAIS-III Symbol Search	***56.78 (8.14)	***56.75 (8.14)	1.97 (0.28)	***26.63 (7.64)	2.09 (0.60)
Trail Making Test A	***11.02 (1.77)	***27.60 (4.44)	*5.70 (0.92)	***7.21 (2.32)	0.20 (0.07)
Colour Trails 1	***42.57 (6.62)	***25.45 (3.96)	0.53 (0.08)	***7.57 (2.36)	0.66 (0.20)
Colour Trails 2	***30.82 (4.70)	***29.08 (4.43)	1.27 (0.19)	***8.97 (2.74)	0.54 (0.16)
Stroop Word Test	0.88 (0.14)	***37.05 (6.06)	*4.13 (0.68)	**5.00 (1.64)	0.29 (0.09)
Stroop Colour Test	***11.78 (1.77)	***18.84 (2.83)	1.08 (0.16)	***14.92 (4.47)	0.33 (0.10)
Stroop Colour-Word Test	***34.59 (5.18)	***25.94 (3.88)	0.37 (0.064)	***21.46 (6.42)	0.23 (0.07)
Halstead Category Test	***40.70 (5.69)	***36.16 (5.05)	***18.32 (2.56)	***25.80 (7.21)	0.80 (0.22)
WCST (Perseverations)	*4.42 (0.79)	**9.26 (1.73)	*5.16 (0.96)	*4.64 (1.73)	0.28 (0.11)
WMS-III Spatial Span	**6.94 (1.10)	***36.98 (5.87)	**1.79 (1.71)	***12.35 (3.92)	0.39 (0.13)
PASAT	3.64 (0.61)	***28.34 (4.74)	0.01 (0.00)	***27.38 (9.15)	*3.54 (1.18)
COWAT (FAS)	0.36 (0.06)	***36.94 (6.46)	1.82 (0.32)	***16.91 (5.91)	0.14 (0.05)
Category Fluency (Animals)	0.10 (0.02)	***58.84 (9.37)	1.20 (0.19)	***19.77 (6.30)	**6.72 (2.14)
Category Fluency (Actions)	0.22 (0.04)	***45.73 (7.20)	0.00 (0.00)	***33.38 (1.50)	***1.72 (3.37)
GPT (Dominant hand)	***50.27 (6.94)	***11.09 (1.53)	**6.91 (0.95)	*4.21 (1.16)	0.12 (0.03)
GPT (Non-dominant hand)	***30.49 (4.98)	2.78 (0.45)	1.72 (0.28)	2.61 (0.85)	0.57 (0.19)

*BVMT-R* Brief Visuospatial Memory Test-Revised; *COWAT* Controlled Oral Word Association Test; *GPT* Grooved pegboard test; *HVLT-R* Hopkins Verbal Learning Test-Revised; *PASAT* Paced Auditory Serial Addition Task; *WAIS-III* Wechsler Adult Intelligence Scale-III; *WCST* Wisconsin Card Sorting Test; *WMS-III* Wechsler Memory Scale-III

Bold indicates largest percentage of variance explained in raw scores on test

*p ≤ 0.05, **p ≤ 0.01, ***p ≤ 0.001

Race, age, and education had strong effects on NC performance. Age accounted for the largest percentage of variance explained in raw scores on tests of visual episodic memory and delayed recall (BVMT-R Total Score: 11.75%, *F*(1, 489) = 79.02,* p* < 0.001; and Delayed Recall: 9.23%,* F*(1, 489) = 59.05,* p* < 0.001), speed of information processing (WAIS-III Symbol Search test: 8.14%,* F*(1, 489) = 56.78,* p* < 0.001), abstraction/executive functions (Colour Trials 1: 6.62%,* F*(1, 489) = 42.57,* p* < 0.001; and 2: 4.70%,* F*(1, 489) = 30.82,* p* < 0.001), and motor function (Grooved Pegboard Test, dominant: 6.94%,* F*(1, 489) = 50.27,* p* < 0.001; and non-dominant hand: 4.98%,* F*(1, 489) = 30.49,* p* < 0.001). The results were all in the expected direction of younger participants performing better.

Education had the strongest effect on raw score variance in tests of verbal episodic memory and delayed recall (HVLT-R Total: 4.52%,* F*(1, 489) = 31.32,* p* < 0.001; and Delayed Recall: 4.39%,* F*(1, 489) = 26.43,* p* < 0.001), measures of processing speed (WAIS-III Digit Symbol test: 14.72%,* F*(1, 489) = 124.84,* p* < 0.001; and Trail Making Test A: 4.44%,* F*(1, 489) = 27.60,* p* < 0.001), attention/working memory (WMS-III Spatial Span: 5.87%,* F*(1, 489) = 36.98,* p* < 0.001), abstraction/executive functions (WCST: 1.73%,* F*(1, 487) = 9.26,* p* < 0.001), reading fluency (Stroop Word Test: 6.06%,* F*(1, 488) = 37.05,* p* < 0.001), and language (COWAT—FAS: 6.46%,* F*(1, 488) = 36.94,* p* < 0.001; the Category Fluency Tests—Animal; 9.37%,* F*(1, 486) = 58.84,* p* < 0.001; and Action/Verb Fluency: 7.20%,* F*(1, 485) = 45.73,* p* < 0.001). Higher education was associated with a better performance.

Race was the strongest predictor of performance on tests of attention/working memory (PASAT: 9.15%,* F*(2, 489) = 27.38,* p* < 0.001) and abstraction/executive functions (Stroop Colour Test: 4.47%,* F*(2, 488) = 14.92,* p* < 0.001; Stroop Colour-Word Test: 6.42%,* F*(2, 488) = 21.46,* p* < 0.001; and the Halstead Category Test: 7.21%,* F*(2, 488) = 25.80,* p* < 0.001).

Minor statistically significant effects of gender were also observed on some of the NC tests. Specifically, women performed better on tests of verbal episodic memory and delayed recall (HVLT-R Total: 0.93%; *F*(1, 489) = 6.45, *p* = 0.011; and Delayed Recall: 1.04%, *F*(1, 489) = 6.26, *p* = 0.013), and one test of processing speed (WAIS-III Digit Symbol test: 1.68%, *F*(1, 489) = 14.24, *p* < 0.001). Men performed better on tests of abstraction/executive functions (Halstead Category Test: 2.56%, *F*(1, 488) = 18.32, *p* < 0.001; and WCST: 0.96%, *F*(1, 487) = 5.16, *p* = 0.023), attention/working memory (WMS III Spatial Span: 1.71%, *F*(1, 489) = 1.79, *p* = 0.001), processing speed (Trail Making Test A: 0.92%, *F*(1, 489) = 5.70, *p* = 0.017), and motor function (Grooved Pegboard Test: dominant hand; 0.95%, *F*(1, 489) = 6.91, *p* = 0.009).

Language of test administration had a significant effect on raw score variance in two tests measuring verbal fluency (Category Fluency Tests – Animal: 2.14%, *F*(2, 486) = 6.72, *p* = 0.001; and Action/Verb Fluency Test: 3.37%, *F*(2, 485) = 10.72, *p* < 0.001), one test of processing speed (WAIS-III Digit Symbol test: 1.00%, *F*(2, 489) = 14.24,* p* = 0.015), and one test of attention/working memory (PASAT: 1.18%, *F*(2, 489) = 3.54, *p* = 0.030).

### Generation of the Prediction Equation

Table 3 summarises the raw score means and standard deviations obtained on each of the NC tests in the norming sample (*n* = 500). These raw scores were converted to normalised scaled scores (*M* = 10; *SD* = 3). The raw-to-scaled score conversions for each NC test are presented in Appendix 1. The formulas used to convert the NC scaled scores to demographically corrected T-scores are presented in Appendix 2.

**Table 3:** Raw test-scores (M, SD) for each NC test in the HNRC battery

NC test	Raw scores		
	Range	*M*	*SD*
BVMT-R (Total)	0–35	19.39	7.27
BVMT-R (Recall)	0–12	7.73	2.80
HVLT-R (Total)	9–34	23.63	4.47
HVLT-R (Recall)	0–12	8.34	2.17
WAIS-III Digit Symbol	15–107	54.42	16.12
WAIS-III Symbol Search	0–50	22.11	8.93
Trail Making Test A	15–147	43.91	19.8
Colour Trails 1	15–162	49.72	2.25
Colour Trails 2	36–293	103.34	4.46
Stroop Word Test	18–128	79.99	17.20
Stroop Colour Test	20–99	58.08	13.37
Stroop Colour-Word Test	5–66	33.45	9.85
Halstead Category Test	6–155	71.70	3.61
WCST (Perseverations)	3–62	13.47	9.91
WMS-III Spatial Span	5–24	13.33	3.59
PASAT	3–49	24.07	1.73
COWAT (FAS)	5–89	29.69	11.91
Category Fluency (Animals)	6–37	14.76	4.42
Category Fluency (Actions)	0–31	11.64	4.54
GPT (Dominant)	46–154	68.75	14.99
GPT (Non-dominant)	52–279	77.31	21.13

*BVMT-R* Brief Visuospatial Memory Test-Revised; *COWAT* Controlled Oral Word Association Test; *GPT* Grooved pegboard test; *HVLT-R* Hopkins Verbal Learning Test-Revised; *PASAT* Paced Auditory Serial Addition Task; *WAIS-III* Wechsler Adult Intelligence Scale-III; *WCST* Wisconsin Card Sorting Test; *WMS-III* Wechsler Memory Scale-III

### Comparison with U.S. Norms

The U.S. T-scores (corrected for age, education, gender, and race) and the newly generated South African T-scores were compared based on the proportions of South African participants defined as “impaired” by each set of normative equations. The U.S. norms for Colour Trials 1 and 2 could not be accessed and were not included this comparison. To estimate the severity of “impairment” in the norming sample, demographically corrected T-scores were converted to deficit scores. Deficit scores across all tests were averaged to compute the Global Deficit Score (GDS). Global impairment was assigned to participants with GDS ≥ 0.50. The impairment rates of the sample as estimated by the U.S. GDS and South African GDS are presented in Table 4.

**Table 4:** NC Test T-scores and Percentage of participants “Impaired” (T < 40) based on U.S. Norms and New South African Norms

Neurocognitive test	U.S. T-score *M *(*SD*)	Impaired by U.S. norms^a^ (%)	Impaired by South African norms^a^ (%)
BVMT-R (Total)	41.9 (9.9)	*40.8	14.8
BVMT-R (Recall)	43.0 (10.8)	*39.4	15.0
HVLT-R (Total)	41.4 (9.1)	*43.0	13.8
HVLT-R (Recall)	44.3 (8.7)	*32.0	14.4
WAIS-III Digit Symbol	39.2 (7.8)	*56.4	14.2
WAIS-III Symbol Search	41.1 (10.0)	*44.0	14.6
Trail Making Test A	38.5 (11.2)	*52.0	14.6
Stroop Word Test	39.2 (10.2)	*50.9	*16.6
Stroop Colour Test	37.7 (10.5)	*61.1	14.4
Stroop Colour-Word Test	39.2 (11.8)	*54.7	15.6
Halstead Category Test	40.0 (11.2)	*50.3	14.6
WCST (Perseverations)	47.8 (11.1)	*24.7	14.7
WMS-III Spatial Span	48.0 (10.4)	*23.4	15.8
PASAT	39.3 (10.1)	*53.8	14.0
COWAT (FAS)	44.3 (10.3)	*32.5	14.8
Category Fluency (Animals)	42.5 (8.9)	*39.0	15.7
Category Fluency (Actions)	40.3(7.9)	*55.6	14.3
GPT (Dominant)	50.2 (10.6)	15.6	13.6
GPT (Non-dominant)	50.7 (10.7)	*16.2	14.8
Global Mean	**42.7 (5.9)**	*62.2	**15.0**

We were unable to obtain U.S norms for Colour Trials 1 and 2. As a result, these tests are not included in the table. The impairment rate for these 2 tests based on South African norms were 12.6% and 14.8% respectively

*BVMT-R* Brief Visuospatial Memory Test-Revised; *COWAT* Controlled Oral Word Association Test; *GPT* Grooved pegboard test; *HVLT-R* Hopkins Verbal Learning Test-Revised; *PASAT* Paced Auditory Serial Addition Task; *U.S.* United States; *WAIS-III* Wechsler Adult Intelligence Scale-III; *WCST* Wisconsin Card Sorting Test; *WMS-III* Wechsler Memory Scale-III

*Impairment rate of > 16%

^a^Impairment rate based on global deficit score derived at a cut-off of ≥ 0.50

The U.S. T-scores generated impairment rates ranging from 15.6% (Grooved Pegboard Test: dominant hand, T-score = 50.2) to 61.1% (Stroop Colour Test, T-score = 37.7). The Grooved Pegboard Test (dominant hand) was the only test that obtained an impairment rate of less than 16%, which is the expected prevalence based upon a 1-SD cut-off for defining “impairment” on individual test measures. More than half of the sample was classified by U.S. norms as impaired by eight of the individual NC tests: the Stroop Colour Test (61.1%); WAIS-III Digit Symbol test (56.4%); Category Fluency Test (Actions) (55.6%); Stroop Colour-Word Test (54.7%); PASAT (53.8%); Trail Making Test A (52.0%); Stroop Word Test (50.9%); and Halstead Category Test (50.3%). A global impairment rate of 62.2% was obtained across tests based on the U.S. GDS. In comparison, the newly developed South African norms generated impairment rates ranging from 13.6% to 16.6%, and the South African GDS evidenced a global impairment rate of 15.0%. When applying the South African norms, the Stroop Word Test was the only test with an impairment rate above 16% (16.6%).

## Discussion

In the present study, South African norms that corrected for age, education, gender, race, and test administration language, were generated for the full HNRC Battery—a comprehensive battery that measures several NC domains sensitive to HIV-related impairment [23]. The HNRC Battery is widely used in international neuroHIV studies and norms for these tests were developed in several LMICs, including Cameroon [45, 77], China [42], Zambia [44], and India [43]. To our knowledge, prior to this study, there were no South African normative data available for the full HNRC Battery.

When age, education, sex, and race-corrected U.S. norms for the HNRC Battery were applied to the performance scores of our sample of neurologically intact, HIV-negative individuals, a high impairment rate of 62.2% was observed. In contrast, using the standard 1-SD cut-off for defining “impairment”, the expected rate of 15.0% was observed for demographically adjusted South African norms. Given that NC tests evaluate abilities that are highly influenced by different historical, cultural, economic, and sociological environments [5, 19, 29, 31, 38], these results were expected and emphasise the need for country-specific NC norms. Similar conclusions were drawn in other international studies reporting significant differences in NC test scores across countries [33, 37, 41, 43, 44, 47].

The present study also identified several demographic effects (i.e., age, education, race, gender, and test administration language) that influenced the NC performance of participants. This finding is in keeping with other norming studies conducted in LMICs [37, 41–47, 77–80]. These demographic characteristics can all potentially influence NC test performance, thereby highlighting the general importance of controlling for demographic characteristics when developing norms for NC measures.

### Demographic Factors in Norm Development

#### Age

Age had the strongest influence on some tests of visual learning and delayed recall (BVMT-R –Total and Recall); processing speed (WAIS-III Symbol Search test); abstraction/executive functions (Colour Trials 1 and 2); and motor functioning (Grooved Pegboard Test—dominant and non-dominant hands), always with younger age being associated with better performance. Verbal fluency was the only NC domain that did not show any significant age effects. These age effects are not surprising as the same effects have been seen on other tests of these constructs. Studies on aging typically found that cognitive change is part of the normal aging process for some NC abilities, such as memory, certain language and visuospatial skills, executive functions, and processing speed [81–85]. The considerable influence of age on NC test performance was reiterated in several norming studies conducted in LMICs, with the general pattern of NC test performance showing a significant decline with increasing age [37, 41, 43–47, 77–80].

#### Education

Also consistent with findings in the U.S. and other international settings, we found that higher education levels were associated with better NC test performance on almost all NC measures. The Grooved Pegboard Test (non-dominant hand), which assesses complex motor function, was the only test without significant education effects. Education best predicted scores on measures of verbal learning and delayed recall (HVLT-R—Total and Recall); processing speed (Trail Making Test A and WAIS-III Digit Symbol test); attention/working memory (WMS-III Spatial Span); executive functions (Stroop Word Colour Test); and verbal fluency. Similar findings were reported in other norming studies conducted in LMICs [37, 41, 43, 44, 46, 77, 78, 80]. To some extent, these findings could reflect the skills developed through formal schooling, although years of formal schooling can reflect other advantages (e.g., SES and quality of education experienced) that are more difficult to quantify. Formal schooling, for example, refines linguistic skills through reading and writing, develops test-wiseness, and reinforces certain values that enhances the learning process, such as the importance of memorising, understanding, and achieving [85, 86]. Further, to the extent that opportunities for higher education are merit-based, more cognitively able youth are likely to eventually complete more education.

It should be noted, however, that the present study provided some control for literacy effects by excluding participants with less than 7 years of formal education. Therefore, the norms developed in this study cannot be generalised to individuals with very low levels of education. In 2017, it was estimated that 13.7% of South Africans aged 20 years or older had no formal education or a formal education of less than 7 years [87].

Furthermore, this study based educational levels on self-reported years of formal education. This does not consider variations in education quality [19, 32, 39]. Hestad et al. [44] controlled for the variation in education quality in Zambia by assessing the formal reading levels of participants using the Zambian Achievement Test (ZAT). The ZAT score contributed significantly to variations in NC test results, above and beyond effect of years of education [44]. Future studies may need to employ similar strategies to control for differences in the quality of education in South Africa and other settings.

#### Gender

We observed minor gender effects. Specifically, women tended to perform somewhat better on measures of verbal learning and delayed recall (HVLT-R—Total and Recall) and processing speed (WAIS-III Digit Symbol test), while men performed better on measures of abstraction/executive functions (Halstead Category test and WCST); attention/working memory (WMS III Spatial Span); processing speed (Trail Making Test A); and motor function (Grooved Pegboard Test—dominant hand). Gender differences in NC functions were reported in several international studies [37, 77, 83, 88, 89] and were associated with genetics [90], functional and structural differences in the brain [90–92], and hormonal influences [90, 93]. However, certain societal and cultural factors, like educational opportunities and expectations, gender equality, and rates of gender-based violence, may also contribute to some of the gender-based variance in NC test performance [44, 94].

#### Race

Ethical considerations surrounding racial-norming in NC measures are widely debated [95]. Ethical challenges regarding demographic groupings based on race include (1) non-discrete socio-political definitions used to categorise racial groups; (2) existence of a large number of potentially different groups within racial categories; (3) existence of racial subcategories that are not psychologically homogeneous; and (4) non-scientific methods used to classify race in research settings, which mostly consist of self-report data [95–97]. Furthermore, the racial effects on NC test performance are likely influenced by complex socio-historical and socio-economic contexts. Therefore, these racial differences may not be globally generalisable and may be country-specific [44]. The development of separate norms could also perpetuate false perceptions regarding the relative abilities of different racial groups [95].

Nonetheless, the use of race as a norming category is highly relevant in the South African context given the country’s history of colonisation and apartheid. Historical policies that disempowered Black and Coloured communities in the apartheid era, has left a legacy of social, economic, and educational inequalities across the South African landscape [98]. High levels of inequality are still apparent across previously oppressed racial groups, even though the post-apartheid government tried to eliminate these inequalities [99–101]. Therefore, for the time being, the impact of these inequalities on NC test performance cannot be ignored. If racial corrections are not applied in local norms, it may result in the misrepresentation of different racial groups and a high rate of misdiagnosis in assessment of NC impairment [73, 95, 97], as highlighted in previous international norming studies [73, 78, 102, 103].

#### Language

Recent South African studies suggested that culturally adapted NC tests can perform equivalently when administered to multilingual adults in either English or isiXhosa [30, 46, 104]. We found that most NC tests did not show significant language effects, but minor language effects were observed on four NC tests. The strongest language effects were observed in two verbal fluency tests, i.e., the Category/Animal and Action/Verb Fluency Tests. Surprisingly, the PASAT-50 (measuring working memory) and WAIS III Digit Symbol test (measuring processing speed) were also significantly influenced by test administration language, even though these tests do not rely on language proficiency.

Furthermore, the present study compared the native language of participants to the language of test administration to see whether language proficiency could account for the variance in test performance. No significant effects were observed, suggesting that NC test performance was not influenced by whether the test was done in the participant’s native language or not. These findings could possibly be attributed to the high exposure of urban South Africans communities to English. English is regarded as the country's lingua franca [105] and is the primary language used in government, business, and commerce [75, 105, 106]. Furthermore, it is widely used in media such as television [106].

Similar to race, language has historical links to inequalities in the South African context [75]. The recent Language-in-Education policy of the South African Department of Education aimed to eliminate these inequalities by promoting multilingualism in schools and developing and promoting native African languages as LoLT [75]. Despite this initiative, most South Africans still prefer English and not their home language as LoLT. These preferences are perpetuated by the belief that English is linked to better education and economic empowerment [75]. More research is needed to better understand the interactions between language, education quality, historical inequalities, and NC test performance in the South African context.

#### Provincial Differences

Data collection was limited to the Cape Metropolitan and Winelands areas in the Western Cape. We found two other South African studies that generated norms for tests that form parts of the HNRC Battery. Robertson et al. [37] generated South African norms for a battery that included six tests from the HNRC Battery [37]. NC data were collected from two South African provinces—Kwazulu-Natal and Gauteng [37]. Van Wijk and Meintjes [79] collected data from several South African provinces to develop norms for the Grooved Pegboard Test [79]. Both studies observed statistically significant regional variances in NC performance. These findings may be attributable to educational inequalities between different municipalities, cultural and socio-economic differences between sites, and different levels of urbanisation [37, 79]. These findings urge caution in the generalisation of normative data across South African provinces.

### Study Limitations

This study has several limitations worth noting. First, the sample size (*n* = 500) was relatively small for a norming study. Research suggests that a normative data-set should include approximately 1 000 participants to minimise the confounding effects of outliers [47, 107]. While our sample size measured up well against other norming studies conducted in LMICs [37, 41–47, 77, 78, 80], the validity of these norms could be improved by the inclusion of a larger sample.

Second, the demographic distribution of the sample was not balanced. Approximately 80% of the sample were women, all participants were recruited from urban areas, and participants with fewer than 7 years of formal education were excluded. Furthermore, the sample was disproportionately young with two-thirds of participants being younger than 35 years of age. Children/adolescents (< 18 years) and older adults (> 65 years) were also excluded. The generalisability of the norms developed here could be improved through the inclusion of a sample with a more proportionate demographic distribution.

Furthermore, no formal literacy tests or reading comprehension tests were used to control for education quality. Variation in the quality of education may result in bias, including that less-literate participants may have struggled to understand and follow NC test instructions [19, 32]. Similarly, no formal tests of English proficiency were conducted even though 79.0% of the HIV-negative sample who completed the NC battery in English were not native English speakers. Language proficiency was judged informally based on the feedback of participants regarding their own language skills and their ability to fluently communicate with study staff in English during recruitment procedures. Future norming studies should aim to assess literacy and language proficiency through formal tests that are valid in their local cultural context.

We were not able to control for all possible confounders without severely compromising the sample size. Variables that could possibly influence NC test performance (e.g., perceived stress and stress reduction habits; dietary habits; exercise habits; and a history of mild head injuries [108–112]) were not controlled for in the present study and is considered a limitation.

However, the same can be said for the stringent exclusion criteria used in the current study, which may limit generalisability to real world PWH samples. Nevertheless, NC test performance is only one component of the process for defining HAND. Test results should be interpreted alongside contextual information regarding the individual’s estimated premorbid functioning, functional impairment, and co-morbidities [113, 114]. Possible co-morbidities and their effect on NC test performance should always be considered.

Finally, data were collected by different data collectors, yet interrater reliability was not measured. Nonetheless, to ensure consistency across assessments, standardised training in the administration of the battery was given to all administrators. All administrators were expected to follow a structured instruction manual verbatim during each assessment and were regularly monitored throughout the study.

## Conclusion

In conclusion, this study provides much-needed South African NC norms that could aid both clinicians and researchers in a wide range of settings in the correct interpretation of NC test results, thereby empowering them to make decisions that are more informed and relevant to therapeutic interventions/pharmacologic treatments. This is especially important in South Africa considering the high HIV prevalence and the high rate of HIV comorbidity in individuals presenting to psychiatric and medical settings. Several demographic factors (i.e., age, education, race, gender, and test administration language) influenced NC performance, highlighting the need to control for demographic characteristics when developing NC test norms. South African norms for the HNRC Battery also differed significantly from published U.S. norms, highlighting the need for localised, country-specific normative data when interpreting NC performance.

## Data Availability

The data that support the findings of this study are available from the corresponding author upon reasonable request.

## Appendix 1

Table for Converting Raw Scores to Scaled Scores

**Table:**

Scaled score	BVMT-R (Total)	BVMT-R (Recall)	HVLT-R (Total)	HVLT-R (Recall)	WAIS-III Digit Symbol	WAIS-III Symbol Search
1	0		0**–**9	0–1	0–15	0
2	1	0	10–11	2	16–19	1–2
3	2–3	1	12–13	3	20–23	3–4
4	4–5	2	14–15	4	24–26	5
5	6–8	3	16		27–31	6–8
6	9–10	4	17–18	5	32–35	9–11
7	11–13	5	19	6	36–40	12–14
8	14–15	6	20–21	7	41–45	15–17
9	16–18	7	22–23	8	46–51	18–20
10	19–21	8	24		52–56	21–23
11	22–23	9	25	9	57–61	24–26
12	24–25	10	26–27	10	62–67	27–29
13	26–28		28		68–72	30–32
14	29–30	11	29–30	11	73–79	33–35
15	31		31		80–86	36–39
16	32	12	32	12	87–92	40–42
17	33–34		33		93–99	43–48
18	35		34		100–104	49
19	36		35–36		105–133	50–60

**Table:**

Scaled score	Trail Making Test A	Colour Trails 1	Colour Trails 2	Stroop Word Test	Stroop Colour Test	Stroop Colour-Word Test
1	15	15	36	18–21	20	5–6
2	16–17	16–19	37–41	22–32	21	7–9
3	18	20–22	42–45	33–39	22–27	10–12
4	19–20	23–24	46–51	40–46	28–35	13–15
5	21–23	25–27	52–56	47–53	36–39	16–18
6	24–25	28–30	57–64	54–60	40–43	19–22
7	26–28	31–33	65–71	61–66	44–47	23–25
8	29–32	34–36	72–79	67–72	48–50	26–28
9	33–36	37–42	80–90	73–78	51–55	29–31
10	37–41	43–48	91–100	79–83	56–60	32–34
11	42–48	49–55	101–112	84–89	61–64	35–37
12	49–56	56–64	113–130	90–95	65–68	38–40
13	57–65	65–72	131–151	96–99	69–73	41–44
14	66–76	73–81	152–174	100–102	74–78	45–49
15	77–91	82–95	175–194	103–109	79–83	50–53
16	92–104	96–110	195–212	110–115	84–90	54–58
17	105–125	111–118	213–261	116–121	91–97	59–61
18	126–142	119–154	262–285	122–126	98	62–65
19	143–147	155–162	286–293	127–128	99	66

**Table:**

Scaled score	Halstead Category Test	WCST (Perseverations)	WMS-III Spatial Span	PASAT	COWAT (FAS)	Category Fluency (Animals)
1	153–155	63–64		0–3	5	
2	146–152	60–62	5		6	
3	136–145	53–59		4	7–9	6
4	125–135	41–52	6	5–6	10–11	7
5	115–124	29–40	7	7–8	12–13	8
6	106–114	22–28	8–9	9–12	14–16	9–10
7	98–105	18–21	10	13–15	17–20	11
8	91–97	15–17	11	16–18	21–23	12
9	81–90	12–14	12	19–21	24–26	13
10	69–80	10–11	13	22–23	27–30	14
11	56–68	8–9	14–15	24–27	31–34	15–16
12	43–55	7	16	28–33	35–38	17–18
13	33–42	6	17	34–39	39–43	19–20
14	24–32	5	18	40–43	44–48	21–22
15	17–23	4	19	44–46	49–54	23
16	11–16		20–21	47	55–60	24–26
17	8–10		22	48	61–66	27–28
18	6–7	2–3	23–24	49	67–82	29–34
19		0–1			83–89	35–37

**Table:**

Scaled score	Category Fluency (Actions)	Grooved Pegboard (Dominant hand)	Grooved Pegboard (Non-dominant hand)
1		154	274–279
2	0–1	146–153	199–273
3	2–3	124–145	142–198
4	4	103–123	114–141
5	5	90–102	103–113
6	6	83–89	94–102
7	7	77–82	86–93
8	8–9	72–76	80–85
9	10	68–71	75–79
10	11–12	65–67	72–74
11	13	62–64	68–71
12	14	59–61	65–67
13	15–16	56–58	63–64
14	17–18	54–55	60–62
15	19–20	52–53	57–59
16	21–23	50–51	55–56
17	24–29	48–49	54
18	30–31	46–47	52–53
19			

*BVMT-R* Brief Visuospatial Memory Test-Revised, *COWAT* Controlled Oral Word Association Test, *GPT* Grooved pegboard test, *HVLT-R* Hopkins Verbal Learning Test-Revised, *PASAT* Paced Auditory Serial Addition Task, *WAIS-III* Wechsler Adult Intelligence Scale-III, *WCST* Wisconsin Card Sorting Test, *WMS-III* Wechsler Memory Scale-III

## Appendix 2

### South African T-scores Corrected for Age, Education, Gender, Race and Language

#### Age/Education/Gender/Race /Language

_Gender:_ _Male__=__0;__Female__=__1_.

_Race—Coloured:_ _No__=__0;__Yes__=__1_.

_Race—White:_ _No__=__0;__Yes__=__1_.

_Language—isiXhosa:_ _No__=__0;__Yes__=__1_.

_Language—Afrikaans:_ _No__=__0;__Yes__=__1_.

**Table:**

Brief Visuospatial Memory Test, Revised (Total)	
50 + 10*[(BVMT-R (Total) scaled score − (0.344 + 4.737*I((education/10)^1^) + 10.762*I((Age/10)^−1^) + 1.111*coloured race + 2.016*white race)/2.557]	
Brief Visuospatial Memory Test, Revised (Recall)	
50 + 10*[(BVMT-R (Recall) scaled score − (1.600 + 4.052*I((education/10)^1^) + 10.084*I((Age/10)^−1^) + 0.455*coloured race + 1.478*white race)/2.805]	
Hopkins Verbal Learning Test, Revised (Total)	
50 + 10*[(HVLT-R (Total) scaled score − (4.478 + 4.342*I((education/10)^1^) + 1.456*coloured race + 3.043*white race − 1.008*isiXhosa language − 0.809*Afrikaans language + 0.722*gender)/2.621]	
Hopkins Verbal Learning Test, Revised (Recall)	
50 + 10*[(HVLT-R (Recall) scaled score − (6.137 + 3.961*I((education/10)^1^) − 0.429*I((Age/10)^1^) + 0.956*coloured race + 2.065*white race − 0.981*isiXhosa language + 0.061*Afrikaans language + 0.801*gender)/2.731]	
Wechsler Adult Intelligence Scale-III Digit Symbol	
50 + 10*[(WAIS-III Digit Symbol scaled score − (4.500 + 6.562*I((education/10)^1^) − 1.041*I((Age/10)^1^) + 2.511*coloured race + 3.859*white race − 0.263*isiXhosa language − 1.070*Afrikaans language + 0.970*gender)/2.194]	
Wechsler Adult Intelligence Scale-III Symbol Search	
50 + 10*[(WAIS-III Symbol Search scaled score − (6.824 + 4.703*I((education/10)^1^) − 0.803*I((Age/10)^1^) + 2.010*coloured race + 4.005*white race − 0.926*isiXhosa language − 0.822*Afrikaans language)/2.288]	
Trail Making Test A	
50 + 10*[(Trail Making Test A scaled score − (6.290 + 4.247*I((education/10)^1^) − 0.443*I((age/10)^1^) + 2.385*coloured race + 3.348*white race − 0.796*gender)/2.547]	
Colour Trails 1	
50 + 10*[(Colour Trails 1 scaled score − (7.923 + 3.594*I((education/10)^1^) − 0.787*I((age/10)^1^) + 2.118*coloured race + 3.887*white race − 0.749*isiXhosa language − 0.744*Afrikaans language)/2.491]	
Colour Trails 2	
50 + 10*[(Colour Trails 2 scaled score − (6.277 + 4.388*I((education/10)^1^) − 0.707*I((age/10)^1^) + 2.378*coloured race + 3.850*white race)/2.484]	
Stroop Word Test	
50 + 10*[(Stroop Word Test scaled score − (4.257 + 4.501*I((education/10)^1^) + 1.613*coloured race + 2.498*white race)/2.710]	
Stroop Colour Test	
50 + 10*[(Stroop Colour Test scaled score − (6.468 + 3.354*I((education/10)^1^) − 0.421*I((age/10)^1) + 2.480*coloured race + 3.860*white race /2.534]	
Stroop Colour-Word Test	
50 + 10*[(Stroop Word-Colour Test scaled score − (7.639 + 3.533*I((education/10)^1^) − 0.737*I((age/10)^1^) + 1.530*coloured race + 3.710*white race)/2.555]	
Halstead Category Test	
50 + 10*[(Halstead Category Test scaled score − (21.534 − 9.080*I((education/10)^−0.5^) − 0.686*I((age/10)^1^) + 1.057*coloured race + 3.989*white race − 0.869*isiXhosa language − 0.633*Afrikaans language − 1.114*gender)/2.366]	
Wisconsin Card Sorting Test (Perseverations)	
50 + 10*[(WCST scaled score − (6.282 + 3.390*I((education/10)^1^) + 0.936*coloured race + 2.727*white race − 0.809*gender)/2.754]	
Wechsler Memory Scale-III Spatial Span	
50 + 10*[(WMS-III Spatial Span scaled score − (6.125 + 4.570*I((education/10)^1^) − 0.377*I((age/10)^1^) + 1.826*coloured race + 3.162*white race − 1.044*gender)/2.562]	
Paced Auditory Serial Addition Test	
50 + 10*[(PASAT scaled score − (5.151 + 4.155*I((education/10)^1^) − 0.027*coloured race + 2.793*white race)/2.639]	
Controlled Oral Word Association Test (FAS)	
50 + 10*[(COWAT scaled score − (4.850 + 3.742*I((education/10)^3^) − 4.855 I((Education/10)^3^*log((Education/10))) + 1.488*coloured race + 3.752*white race)/2.566]	
Category Fluency: Animals	
50 + 10*[(Category Fluency: Animals scaled score − (3.446 + 5.256*I((education/10)^1^) + 2.599*coloured race + 3.676*white race − 0.452*isiXhosa language − 1.058*Afrikaans language)/2.410]	
Category Fluency: Action	
50 + 10*[(Category Fluency: Action scaled score − (4.260 + 4.580*I((education/10)^1^) + 1.823*coloured race + 4.336*white race + 0.730*isiXhosa language − 1.604*Afrikaans language)/2.473]	
Grooved Pegboard Test (Dominant hand)	
50 + 10*[(GPT: Dominant hand scaled score − (11.837 − 0.815*I((age/10)^1^) + 0.907*coloured race + 3.131*white race − 0.566*isiXhosa language − 0.632*Afrikaans language + 0.627*gender)/2.795]	
Grooved Pegboard Test (Non-dominant hand)	
50 + 10*[(GPT: Non-dominant hand scaled score − (8.251 + 2.897*I((education/10)^1^) − 0.938*I((age/10)^1^) + 1.280*coloured race + 2.701*white race + 1.117*gender)/2.696]	
